# Updated Estimates and Mapping for Prevalence of Chagas Disease among Adults, United States

**DOI:** 10.3201/eid2807.212221

**Published:** 2022-07

**Authors:** Amanda Irish, Jeffrey D. Whitman, Eva H. Clark, Rachel Marcus, Caryn Bern

**Affiliations:** University of California–San Francisco, San Francisco, California, USA (A. Irish, J.D. Whitman, C. Bern);; Baylor College of Medicine, Houston, Texas, USA (E.H. Clark);; Medstar Union Memorial Hospital and Latin American Society of Chagas, Washington, DC, USA (R. Marcus)

**Keywords:** Chagas disease, *Trypanosoma cruzi*, epidemiology, parasites, United States

## Abstract

We combined American Community Survey data with age-specific *Trypanosoma cruzi* prevalence derived from US surveys and World Health Organization reports to yield estimates of Chagas disease in the United States, which we mapped at the local level. In addition, we used blood donor data to estimate the relative prevalence of autochthonous *T. cruzi* infection. Our estimates indicate that 288,000 infected persons, including 57,000 Chagas cardiomyopathy patients and 43,000 infected reproductive-age women, currently live in the United States; 22–108 congenital infections occur annually. We estimated ≈10,000 prevalent cases of locally acquired *T. cruzi* infection. Mapping shows marked geographic heterogeneity of *T. cruzi* prevalence and illness. Reliable demographic and geographic data are key to guiding prevention and management of Chagas disease. Population-based surveys in high prevalence areas could improve the evidence base for future estimates. Knowledge of the demographics and geographic distribution of affected persons may aid practitioners in recognizing Chagas disease.

## Abstract 2

Geographic scale estimates of disease in older Latin America–born US residents may be useful for prevention and early recognition of chronic sequelae.

Six million persons are estimated to have Chagas disease in the Americas; 20%–30% of those cases will progress to cardiac or gastrointestinal disease (*1*). Early treatment of infection with the causative parasite, *Trypanosoma cruzi*, provides the best chance to decrease progression risk; cure rates are >60% in those treated as children (*2*,*3*). Cure rates among adults are unclear; the accepted test of cure is reversion to negative serologic test results, which requires years to decades, and the time to negative serologic results is inversely proportional to the duration of infection (*4*). Because the date of *T. cruzi* infection is nearly always unknown, age is commonly used as a proxy for duration. Infected persons are typically asymptomatic for decades. In those with established Chagas cardiomyopathy, antiparasitic treatment is unlikely to alter heart disease progression (*5*). Thus, early, active screening during the asymptomatic period is essential to achieve timely diagnosis and effective treatment. Since the establishment of regional control programs in the 1990s, many Latin America countries have mounted community- and facility-based programs, most commonly focused on screening of children and pregnant women (*6*,*7*). No such large-scale programs exist in the United States.

Enzootic transmission by local triatomine species occurs across the southern United States from coast to coast; Lynn et al. summarized 76 suspected or confirmed autochthonous human *T. cruzi* infections (*8*). However, locally acquired infections are vastly outnumbered by those acquired by immigrants from Latin America in their countries of origin before arrival in the United States. No nationally representative *T. cruzi* prevalence data exist for the United States; disease burden estimates have been based on reported national prevalence figures from Latin America countries. These estimates suggest that 240,000–350,000 US residents of Latin America origin may have *T. cruzi* infection (*9*). However, infection rates are heterogeneous within countries, so national-level prevalence estimates may not reflect prevalence among US immigrants.

Calls for more widespread screening and diagnostic testing for Chagas disease in the United States are growing (*10*–*12*). Finer-scale geographic data would be of great help in the targeting of such efforts. Local screening of at-risk populations in Los Angeles, California; the District of Columbia; and the Boston, Massachusetts, metropolitan areas provide a more accurate reflection of prevalence in some US populations (*13*–*15*). Using data from the American Community Survey (ACS) (*16*), we developed new age-structured estimates and interactive maps of Chagas disease prevalence at the local level. We present these data to support geographic targeting of screening efforts and setting priorities for healthcare providers and public health outreach to address Chagas disease in the United States.

## Methods

### Prevalence by Age and Country of Origin

Because *T. cruzi* infection is lifelong in the absence of effective antiparasitic treatment, the prevalence of infection tends to rise as age increases (*17*). Those patterns may also reflect improved vector control for patients who grew up more recently in endemic settings compared with those in older age cohorts (*17*); also, age is used as a determinant for treatment recommendations (*1*). Together, these issues make age-structured estimates crucial to public health efforts. Past estimates have relied on aggregate prevalence figures derived from data provided by member countries and published by the World Health Organization (*18*). For our estimates, we used *T. cruzi* seroprevalence data from US populations to the greatest extent possible (*13*–*15*). Data are available for immigrants from the most frequent Chagas disease–endemic countries of origin: Mexico, El Salvador, Guatemala, Honduras, and Colombia. In addition, data are available from a metropolitan area with a high number of immigrants from Bolivia, a group that contributes disproportionately to the Chagas disease burden because of very high prevalence in some regions of Bolivia (*13*). Data for children <18 years of age are extremely sparse. One of the screening studies that underpin our assumptions included 225 children, of whom none were infected (*14*). Those data were insufficient to obtain a reliable estimate for children; for that reason, our estimates are for adults only.

We used the age-specific pattern for El Salvador in US survey data to model prevalence patterns for immigrants from other countries of origin. Although more immigrants to the United States are from Mexico than El Salvador, *T. cruzi* prevalence is substantially higher among those from El Salvador (*13*,*14*,*19*,*20*); for this reason, the patterns were clearer and the age-stratified estimates more stable for immigrants from El Salvador. The general finding of prevalence increasing with age holds true in data from immigrants from Latin America in the United States (*13*–*15*), as well as in surveys from urban and rural areas of Latin America (*21*–*23*). We then calculated the ratio of the overall prevalence in persons from a given country to the prevalence for immigrants from El Salvador. We multiplied this country-level correction factor by the El Salvador estimates to yield estimated age-specific prevalence for immigrants from each country (Appendix 1 Table 1). For Mexico, Guatemala, Honduras, Colombia and Bolivia, we derived the correction factor from the mean of estimated prevalence from US surveys plus the WHO estimate; for all other countries of origin, we used WHO estimates (*18*).

### Estimates of Foreign-Born Population by Age Group and Public Use Micro-Area 

The ACS is an annual survey conducted to supplement the decennial census (*16*). We used the 5-year data, based on a 5% sample of the US population, because they provide the most statistically reliable estimates, a particular concern for this study because we calculated estimates for small population subgroups at the public use micro-area (PUMA) level for mapping. PUMAs partition states into areas containing >100,000 residents and are the smallest geographic area for which complete microdata are available. Because not all counties can be characterized using PUMA data, we could not map at the county level. Estimates are interpreted as period estimates (e.g., the Chagas disease prevalence in 2014–2018).

We extracted relevant microdata for 2014–2018 from IPUMS-USA, which collects and harmonizes data from the census and ACS (Appendix 1). Using these data, we estimated the overall adult population and population of adult Latin America–born US residents by country of origin and age group (Appendix 1 Table 2). 

### Estimates of the Clinical Burden of Chagas Disease in the United States

We used the infection prevalence and population figures to calculate the prevalence of Chagas disease at the PUMA level for mapping and national level for summary estimates. We produced estimates of the number of patients with Chagas cardiomyopathy in the United States by applying age-specific cardiomyopathy prevalence rates among *T. cruzi*–infected persons in population-based studies from disease-endemic countries to our US infection estimates by age group (*24*–*26*). 

We estimated the risk for congenital transmission in the United States using age-specific infection prevalence and birth rate statistics. To estimate age-specific birth rates among foreign-born women from Latin America, we started with the reported number of live births per 1,000 Hispanic women by age group in 2017 (*27*). That figure includes women of Hispanic origin born in the US as well as women born in Latin America. We therefore multiplied by a correction factor of 1.22 to adjust for the higher birth rate among US resident women born in Latin America (82.3) compared with all Hispanic women (67.6) (*27*,*28*). We then applied a range of vertical transmission rates of 1%–5% to estimate a likely range for the number of congenitally infected infants born in 2017. In a recent meta-analysis, the estimated vertical transmission rate for *T. cruzi*–infected women in nonendemic countries was 2.7%, falling within the range we used (*29*). However, most of the data in the meta-analysis came from immigrants from Bolivia in Spain. Data for women from Mexico and Central America are extremely sparse, and we felt the uncertainty expressed by the range was more appropriate than a single point estimate.

Finally, we calculated the relative number of locally acquired autochthonous *T. cruzi* infections in the United States, based on estimates that 5.5%–7.5% of blood donor infections were locally acquired (*30*). We corrected for underrepresentation of Hispanic populations in donor data (*31*).

### Statistical Analysis and Mapping

We performed analyses in R version 4.0.4 (https://www.r-project.org). We obtained point estimates and 95% CIs using person-level replicate weights. We generated interactive, web-based maps to display estimates for the total number of infected adults and the prevalence of Chagas disease in the total population and in the Latin America–born population at the PUMA level (Appendix 1). 

## Results

We estimated that 287,711 adult Latin America–born US residents were living with Chagas disease during the period 2014–2018 (Table 1). Of those, 68% (196,907) were >50 years of age; case numbers were low in younger age groups. The marked age dependence of both *T. cruzi* infection prevalence and Chagas cardiomyopathy indicates that >85% of the estimated 57,000 Chagas cardiomyopathy cases occur in those >50 years of age (Table 2). Because prevalence among women of childbearing age is relatively low, we estimate relatively few congenital infections (Table 3). On the basis of blood donor data, we estimated as many as 10,000 locally acquired *T. cruzi* infections in the United States (Appendix Table 3).

**Table 1:** Estimates of the number of Latin America–born adults with Chagas disease in the United States

Birth country	*Trypanosoma cruzi* infection prevalence, %	Estimated no. infected adults by age group			
		All ages	18–34	35–49	>50
Argentina	3.64	14,463	600	2,592	11,271
Belize	0.33	344	15	53	276
Bolivia	18.3	27,335	1,650	5,262	20,423
Brazil	0.61	3,865	379	1,049	2,437
Chile	0.70	1,560	69	226	1,265
Colombia	0.51	7,840	398	1,260	6,182
Costa Rica	0.17	289	18	55	216
Ecuador	1.38	11,200	719	2,316	8,165
El Salvador	1.90	41,788	3,287	11,260	27,241
Guatemala	1.13	14,143	1,846	4,109	8,188
Guyana, French Guiana, Suriname	0.84	5,171	183	746	4,242
Honduras	0.65	5,208	671	1,606	2,931
Mexico	0.73	141,554	10,730	36,413	94,411
Nicaragua	0.52	2,773	131	528	2,114
Panama	0.52	1,810	64	233	1,513
Paraguay	2.13	679	75	134	470
Peru	0.44	4,125	192	728	3,205
Uruguay	0.24	234	11	39	184
Venezuela	0.71	3,330	315	842	2,173
All Latin America countries	1.64	287,711	21,353	69,451	196,907

**Table 2:** Estimated Latin America–born persons with Chagas cardiomyopathy in the United States

Age, y	No. infected	No. (%) with Chagas cardiomyopathy
18–34	21,353	854 (4)
35–49	69,451	6,945 (10)
>50	196,907	49,227(25)
All ages	287,711	57,027 (19.8)

**Table 3:** Estimated annual births to *Trypanosoma* *cruzi*–infected women and congenital infections, United States

Maternal age, y	No. women infected	Live births/1,000 women*	No. births to infected women	No. infected infants/y	
				Lower limit, 1%	Upper limit, 5%
18–19	683	64.3	44	0	2
20–24	2,134	114.4	244	2	12
25–29	3,051	136.4	416	4	21
30–34	3,933	117.6	463	5	23
35–39	11,553	66.6	770	8	38
40–44	11,573	17.7	205	2	10
45–49	10,356	1.2	13	0	1
All ages	43,283		2,154	22	108

*Age-specific birth rates for all Hispanic women in 2017 multiplied by 1.22 to correct for higher birth rates among foreign-born Hispanic women (see Methods).

The PUMA-level maps illustrate the marked geographic heterogeneity of estimated *T. cruzi* infection prevalence and the burden of Chagas disease in the United States (https://amandairish.github.io/chagas_maps). Foci of high disease burden vary substantially in demography, geography and healthcare access, as we saw in the Houston, Texas, metropolitan area (Appendix 2); in southern California (Appendix 3); and in the Washington, DC, metropolitan area (Appendix 4). The metropolitan areas with the highest number of estimated Chagas disease cases reflect major population centers, whereas areas with the highest percentage of infected residents include midsized cities in states with a high proportion of Latin America–born residents (Table 4).

**Table 4:** US metropolitan areas with the highest estimated prevalence of Chagas disease

Location	*Trypanosoma cruzi*–infected adults	Prevalence in total adult population, %	Prevalence in Latin America–born adult population, %
Top 10 in total number of *T. cruzi–*infected adults			
Los Angeles-Long Beach-Anaheim, CA	44,768	0.43	1.97
New York-Newark-Jersey City, NY-NJ-PA	28,304	0.18	1.89
Washington-Arlington-Alexandria, DC-VA-MD-WV	17,745	0.38	3.85
Miami-Fort Lauderdale-West Palm Beach, FL	15,586	0.32	1.93
Houston-The Woodlands-Sugar Land, TX	14,175	0.29	1.60
Riverside-San Bernardino-Ontario, CA	11,070	0.33	1.71
Chicago-Naperville-Elgin, IL-IN-WI	10,931	0.15	1.51
Dallas-Fort Worth-Arlington, TX	9,887	0.19	1.37
San Francisco-Oakland-Hayward, CA	6,898	0.18	1.76
San Diego-Carlsbad, CA	5,730	0.22	1.54
Top 10 in overall *T. cruzi* prevalence			
El Centro, CA	956	0.74	1.76
Laredo, TX	1,025	0.57	1.49
McAllen-Edinburg-Mission, TX	3,193	0.56	1.49
El Paso, TX	3,387	0.56	1.77
Brownsville-Harlingen, TX	1,564	0.54	1.66
Yuma, AZ	738	0.48	1.56
Los Angeles-Long Beach-Anaheim, CA	44,768	0.43	1.97
Salinas, CA	1,503	0.41	1.35
Merced, CA	756	0.40	1.46
Washington-Arlington-Alexandria, DC-VA-MD-WV	17,745	0.38	3.85

## Discussion

To address Chagas disease in the United States, public health practitioners and healthcare providers need to know where and among whom to target their efforts. Our updated estimates define the demographics and provide a detailed geography of Chagas disease. In data from both the United States (*13*–*15*) and Chagas disease–endemic countries (*21*–*23*), the infection prevalence increases with increasing age. The use of prevalence and age structure assumptions based on data from several US populations of interest make these new estimates a more accurate reflection of *T. cruzi* infection and illness than previous calculations (*9*,*32*). By mapping the resulting data at the most local level possible, we have constructed interactive maps that enable providers to assess risk in their catchment area (*16*). Such maps could be developed to target screening efforts for other conditions for which migrants bear a disproportionate risk (*33*).

These new estimates add nuance to the already complex landscape of efforts to address Chagas disease (*1*,*34*). Our updated estimate of ≈288,000 *T. cruzi*–infected US residents is consistent with earlier figures of ≈240,000 to ≈350,000 (*9*,*32*). However, our new age-structured estimates indicate that two thirds of persons with Chagas disease in the United States are >50 years of age. This finding substantially increases the estimate of patients with Chagas cardiomyopathy (57,000 in our estimates vs. 30,000–45,000 in the 2009 estimates) and decreases the projected number of annual congenital *T. cruzi* infections (22–108 in our data vs. 63–315 in 2009 data) (*32*).

Antitrypanosomal treatment recommendations are strongest for younger age groups, based on the more robust data for benefit among children than adults (*35*,*36*). In the United States, as in Latin America, at-risk women of reproductive age should be screened for Chagas disease, to offer them treatment and detect infected infants early in life (*36*,*37*). Treatment of women before pregnancy is associated with an estimated 95% decrease in risk for subsequent congenital transmission (*4*,*38*). We were unable to make a disease burden estimate for children <18 years of age; 1 of the 3 US studies used to underpin the estimates included children, none of whom was infected (*14*). Children in the United States are also at risk if they were born to women with Chagas disease; hundreds of US-born children <18 are probably living with undetected *T. cruzi* infection acquired at birth. Maternal birthplace is, therefore, a crucial piece of information to assess risk among US-born persons with roots in Latin America.

Persons with Chagas cardiomyopathy also benefit from accurate and timely diagnosis. Clinical trial data have failed to show substantial effects of antitrypanosomal therapy on progression of established Chagas cardiomyopathy, reinforcing the urgency to institute active screening to detect infections before cardiac damage occurs (*5*,*39*). Nevertheless, good cardiac management substantially improves survival and quality of life, and the United States has the resources to appropriately evaluate and manage every infected patient (*40*). Patients who receive cardiac transplants for end-stage Chagas cardiomyopathy have a survival rate equivalent to or better than that of patients who receive transplants for other etiologies, as long as the infection is recognized and the patient actively monitored for reactivation (*41*–*43*). Pretransplant diagnosis of *T. cruzi* infection is crucial to ensure good outcomes (*41*).

Our estimates improve on previous efforts (*9*,*32*) but suffer from some of the same limitations in the empirical data underpinning their assumptions. US data were available from 3 metropolitan areas (*13*–*15*), and data for children were extremely sparse. The US data were based on clinical screening and community convenience samples, not population-based sampling. The results may be affected by differences in access to care, catchment areas, and awareness among participants. ACS datasets lack the data needed to make estimates for some counties, including several of those comprising the highest-burden PUMAs. Thus, we were unable to show a county-level map, which might have been useful for public health targeting. We have no direct data for the incidence of congenital *T. cruzi* transmission in the United States. Only 2 congenital infections have been reported, both with moderately severe manifestations (*44*,*45*). In the absence of screening, most infected infants with minimal or no symptoms were undoubtedly missed. Because of the indirect calculation method, and because foreign-born donors may have been less likely than US-born donors to participate in the donor follow-up study (*30*), our estimate for locally acquired Chagas disease provides an indication of the relative order of magnitude of this problem and may represent an overestimate.

Effectively addressing Chagas disease is complicated by the heterogeneity of healthcare systems in the United States. States play a major role in determining services for the indigent, uninsured, and undocumented persons who are at highest risk for Chagas disease, so there is no universal pathway for these persons to receive affordable healthcare (*46*). Nevertheless, most states have programs to cover uninsured pregnant women, infants, and young children. Thus, prenatal testing and evaluation of newborns and older children of infected women constitute high-priority, cost-effective aspects of Chagas disease control that should be within our immediate reach (*11*,*12*). Managing the chronic sequelae of Chagas disease is complex and costly, and access to such care for uninsured patients varies widely from state to state. Federally qualified health centers may lack the capacity to provide access to specialty services such as infectious diseases, cardiology, and gastroenterology (*47*). Strategies to enhance awareness among relevant providers, including primary care physicians, obstetricians, cardiologists and gastroenterologists, are urgently needed. Targeting locations with the highest Chagas disease burden will improve screening, management and health care access (*48*).

Early treatment has the potential to prevent congenital transmission and decrease the future burden of cardiomyopathy and other chronic sequelae of Chagas disease. Screening of asymptomatic persons at epidemiologic risk will be essential to achieve these goals (*12*). Population-based surveys in high-prevalence areas could identify those eligible for treatment, and at the same time, greatly improve the evidence base for future estimates. However, such surveys would be much more resource intensive than screening in primary-healthcare settings. Early recognition of Chagas cardiomyopathy is equally necessary to guide accurate medical and surgical management to improve quality of life and survival. Many of those at highest risk for COVID-19 include the target populations identified in our Chagas disease estimates, and the outreach methods and community partnerships crucial to the response to the pandemic provide a potential template for addressing Chagas disease (*49*).

Appendix 1 Additional information about Chagas disease in the United States.

Appendix 2 Additional information about Chagas disease in the Houston, Texas, metropolitan area.

Appendix 3 Additional information about Chagas disease in Southern California.

Appendix 4 Additional information about Chagas disease in the Washington, DC, metropolitan area.
