# Supplementary material for: Updated Estimates and Mapping for Prevalence of Chagas Disease among Adults, United States
Source: Emerg Infect Dis. 2022 Jul;28(7):1313–20. doi: 10.3201/eid2807.212221 (PMC9239882; doi:10.3201/eid2807.212221)
Supplement: Appendix 1 — Additional information about Chagas disease in the United States. [file 21-2221-Techapp-s1.pdf]

Article DOI: https://doi.org/10.3201/eid2807.212221

Updated Estimates and Prevalence of Chagas Disease among Adults, United States

Appendix 1

Statistical Methods

We extracted relevant microdata for 2014–2018 from IPUMS-USA (*1*). We used the 5-year data, based on a 5% sample of the US population, because they provide the most statistically reliable estimates (*2*). We performed analyses in R version 4.0.4 (*3*) and RStudio version 1.4.1106 (*4*). Data were read into R with the ipumsr package (*5*). Point estimates and 95% confidence intervals were obtained using the tidyverse (*6*) and srvyr (*7*), with person-level replicate weights (*8*). We obtained the 2018 TIGER/Line shapefiles for PUMAs and states from the US Census Bureau using the tigris package (*9*) and created maps using the tmap package in R (*10*).

Interactive Maps

Interactive maps are available at https://amandairish.github.io/chagas_maps.

Map 1 shows the estimated total number of adult Latin American-born residents with Chagas disease by Public Use Micro Area (PUMA). PUMAs are determined by the US Census bureau and divide states into areas containing ≥100,000 residents. Chagas disease burden estimates are based on number of foreign-born Latin American immigrants (calculated using American Community Survey 2014–2018 data) and estimated *Trypanosoma cruzi* infection prevalence in their countries of origin.

Map 2 shows estimated prevalence of *Trypanosoma cruzi* infection in the overall adult population by Public Use Micro Area (PUMA). PUMAs are determined by the US Census bureau and divide states into areas containing ≥100,000 residents. Chagas disease burden

estimates are based on number of foreign-born Latin American immigrants (calculated using American Community Survey 2014–2018 data) and estimated *Trypanosoma cruzi* infection prevalence in their countries of origin.

Map 3 shows estimated prevalence of *Trypanosoma cruzi* infection among adult Latin American-born residents by Public Use Micro Area (PUMA). PUMAs are determined by the US Census Bureau and divide states into areas containing ≥100,000 residents. Chagas disease burden estimates are based on number of foreign-born Latin American immigrants (calculated using American Community Survey 2014–2018 data) and estimated *Trypanosoma cruzi* infection prevalence in their countries of origin.

References

1. Ruggles S, Flood S, Foster S, Goeken R, Pacas J, Schouweiler M, et al. IPUMS USA: Version 11.0, 2014–2018 5-year dataset. Minneapolis, MN: IPUMS, 2021; 2021.

2. U.S. Census Bureau. Understanding and using ACS single-year and multiyear estimates. understanding and using American Community Survey data: what all data users need to know. Washington, D.C.: U.S. Census Bureau; 2020. p. 13–6.

3. R Core Team. R: A language and environment for statistical computing. Vienna, Austria: R Foundation for Statistical Computing; 2021.

4. R Studio Team. RStudio: Integrated development environment for R. Boston, MA: RStudio, PBC 2021.

5. Freedman Ellis G, Burk D. ipumsr: Read 'IPUMS' extract files. R package version 0.4.5. 2020.

6. Wickham H, Averick M, Bryan J, Chang W, D'Agostino McGowan L, François R, et al. Welcome to the tidyverse. J Open Source Softw. 2019;4:1686. https://doi.org/10.21105/joss.01686

7. Freedman Ellis G, Schneider B. srvyr: 'dplyr'-like syntax for summary statistics of survey data. R package version 1.0.0.; 2020.

8. IPUMS USA. Replicate weights in the American Community Survey / Puerto Rican Community Survey. Minneapolis, MN: IPUMS, 2021.

9. Walker K. tigris: load census TIGER/line shapefiles. R package version 1.0.; 2020. https://cran.r-project.org/web/packages/tigris

10. Tennekes M. tmap: thematic maps in R. J Stat Softw. 2018;84:1–39. https://doi.org/10.18637/jss.v084.i06

Appendix Table 1. Estimated *T. cruzi* infection prevalence by country of origin and age group. See methods section for derivation.

			Age-specific *T. cruzi* prevalence		
Country of origin	Overall prevalence	Correction factor	18–34 y	35–49 y	≥50 y
Argentina	3.64%	1.916	1.68%	4.15%	13.05%
Belize	0.33%	0.174	0.15%	0.38%	1.18%
Bolivia	18.30%	9.629	8.45%	20.86%	65.60%
Brazil	0.61%	0.319	0.28%	0.69%	2.17%
Chile	0.70%	0.368	0.32%	0.80%	2.51%
Colombia	0.51%	0.268	0.23%	0.58%	1.82%
Costa Rica	0.17%	0.089	0.08%	0.19%	0.61%
Ecuador	1.38%	0.726	0.64%	1.57%	4.95%
El Salvador	1.90%	1.000	0.88%	2.17%	6.81%
Guatemala	1.13%	0.596	0.52%	1.29%	4.06%
Guyana, French Guiana, Surinam	0.84%	0.442	0.39%	0.96%	3.01%
Honduras	0.65%	0.340	0.30%	0.74%	2.32%
Mexico	0.73%	0.385	0.34%	0.83%	2.63%
Nicaragua	0.52%	0.275	0.24%	0.60%	1.87%
Panama	0.52%	0.271	0.24%	0.59%	1.85%
Paraguay	2.13%	1.121	0.98%	2.43%	7.64%
Peru	0.44%	0.231	0.20%	0.50%	1.58%
Uruguay	0.24%	0.125	0.11%	0.27%	0.85%
Venezuela	0.71%	0.374	0.33%	0.81%	2.55%

Appendix Table 2. Estimated number of Latin American-born US residents by country of origin

	Adults ≥18 yr		18–34 y		35–49 y		≥ 50 y	
Birth country	Total N	95% CI	N	95% CI	N	95% CI	N	95% CI
Argentina	184,510	177,552–191,468	35,712	32,720–38,704	62,464	59,144–65,784	86,334	82,779–89,889
Belize	47,446	44,356–50,536	10,110	8,668–11,552	14,033	12,465–15,601	23,303	21,368–25,238
Bolivia	75,889	71,417–80,361	19,529	17,459–21,599	25,228	22,994–27,462	31,132	28,877–33,387
Brazil	399,218	388,511–409,925	135,402	130,279–140,525	151,720	146,574–156,866	112,096	107,495–116,697
Chile	100,100	95,402–104,798	21,361	19,078–23,644	28,345	25,924–30,766	50,394	47,476–53,312
Colombia	726,029	710,572–741,486	169,422	163,182–175,662	217,417	210,243–224,591	339,190	331,399–346,981
Costa Rica	86,883	82,715–91,051	23,159	21,103–25,215	28,229	25,907–30,551	35,495	33,241–37,749
Ecuador	425,100	414,195–436,005	112,859	108,143–117,575	147,228	140,736–153,720	165,013	159,920–170,106
El Salvador	1,294,479	1,272,024–1,316,934	374,741	364,324–385,158	519,878	509,271–530,485	399,860	391,308–408,412
Guatemala	872,513	856,267–888,759	352,905	342,106–363,704	318,089	310,154–326,024	201,519	195,831–207,207
Guyanas*	266,182	258,629–273,735	47,182	44,030–50,334	78,002	74,650–81,354	140,998	135,985–146,011
Honduras	569,429	555,415–583,443	224,929	217,648–232,210	218,041	211,012–225,070	126,459	121,326–131,592
Mexico	11,132,323	11,063,940–11,200,706	3,173,938	3,145,094–3,202,782	4,362,499	4,336,805–4,388,193	3,595,886	3,564,495–3,627,277
Nicaragua	255,406	247,735–263,077	54,067	51,032–57,102	88,594	84,418–92,770	112,745	107,629–117,861
Panama	148,514	143,656–153,372	26,971	24,918–29,024	39,668	37,265–42,071	81,875	78,176–85,574
Paraguay	19,310	17,484–21,136	7,623	6,531–8,715	5,528	4,494–6,562	6,159	5,231–7,087
Peru	443,222	433,066–453,378	94,702	90,614–98,790	145,258	140,158–150,358	203,262	197,841–208,683
Uruguay	45,755	42,665–48,845	9,703	8,256–11,150	14,485	12,842–16,128	21,567	19,729–23,405
Venezuela	285,401	276,177–294,625	96,023	91,291–100,755	104,050	99,029–109,071	85,328	81,466–89,190
All	17,377,709	17,292,099–17,463,319	4,990,338	4,951,703–5,028,973	6,568,756	6,538,508–6,599,004	5,818,615	5,783,539–5,853,691

*Guyana, French Guiana and Suriname

Appendix Table 3. Estimate of locally acquired *T. cruzi* infections*

Steps in calculation	No.	Derivation
Seropositive blood donors 2007–2019	2,462	AABB data
Estimated % locally acquired	6.50	Mean of 5.5% and 6.5% (Cantey 2012)
Estimated number of locally acquired donor infections	160	6.5% x 2462
Ratio infections in foreign born donors to locally acquired	14.38	(2462–160) / 160
Ratio doubled because Hispanics donate at 50% of the rate of non-Hispanic	28.77	14.38 × 2 (Murphy et al 2009)
Estimated infections among Latin American born	287,711	Table 1
Estimated locally acquired infections	10,000	287,711 divided by 28.77

*AABB, Association for the Advancement of Blood & Biotherapies

Sources: Cantey PT, Stramer SL, Townsend RL, et al. The United States Trypanosoma cruzi Infection Study: evidence for vector-borne transmission of the parasite that causes Chagas disease among United States blood donors. Transfusion 2012; 52 (*9*): 1922–30.

Murphy EL, Shaz B, Hillyer CD, et al. Minority and foreign-born representation among U.S. blood donors: demographics and donation frequency for 2006. Transfusion 2009; 49 (*10*): 2221–8.
