# Supplementary material for: Updated Estimates and Mapping for Prevalence of Chagas Disease among Adults, United States
Source: Emerg Infect Dis. 2022 Jul;28(7):1313–20. doi: 10.3201/eid2807.212221 (PMC9239882; doi:10.3201/eid2807.212221)
Supplement: Appendix 2 — Additional information about Chagas disease in the Houston, Texas, metropolitan area. [file 21-2221-Techapp-s2.pdf]

Article DOI: https://doi.org/10.3201/eid2807.212221

Updated Estimates and Prevalence of Chagas Disease among Adults, United States

Appendix 2

Chagas Disease in Houston, Texas

Texas is one of 7 states in which Chagas disease is a notifiable condition (*1*). Between 2013 and 2019, 2 acute and 32 chronic locally-acquired *T. cruzi* infections were reported to the Texas Department of State Health Services; the majority of the chronic infections were detected via blood donor screening (*2*). Despite the focus on local transmission in state reporting, infections acquired in Latin America substantially outnumber autochthonous infections and most of the reports come from the 3 largest metro areas, San Antonio, Dallas, and Houston.

Houston is the largest city in Texas and is located in Harris County. Of Houston's 6.7 million persons, 14% or nearly 1 million, were born in Latin America; we estimate that 14,000 of these immigrants have Chagas disease, nearly 3,000 have Chagas cardiomyopathy and 135 infected women give birth to infants at risk for congenital Chagas disease each year. A study of women who delivered in a large public hospital in Houston found that 0.25% had *T. cruzi* infection, all from Mexico or Central America (*3*). Mapping at Public Use Micro-Area (PUMA) level confirms a heterogeneous distribution of infected individuals, with Latin American immigrant populations and projected *T. cruzi* infections concentrated in several quadrants inside the 610 freeway loop (Figure).

As elsewhere in the United States, Chagas disease diagnostic testing is underutilized in Harris County. Most people with Chagas disease are unaware of their disease, and most US clinicians do not know that they can or should test for *T. cruzi* infection (*4*,*5*). The presence of significant local transmission cycles in Texas further complicates decision-making (*1*). Harris County residents who are un- or underinsured can receive healthcare from a well-organized safety net health system (the Harris Health System), regardless of immigration status. However,

many eligible persons are unaware that this system exists or afraid to access it due to fear of increasing their risk for deportation. Additional impediments to access include the inability to take time from work, lack of transportation and language barriers. Finally, given the high proportion of infections of Mexican origin, up to 10% may be missed due to relatively low sensitivity of currently available diagnostic tests (*6*).

References

1. Bern C, Messenger LA, Whitman JD, Maguire JH. Chagas disease in the United States: a Public Health Approach. Clin Microbiol Rev. 2019;33:e00023-19. PubMed https://doi.org/10.1128/CMR.00023-19

2. Texas Department of State Health Services. Chagas disease data. 2021 May 4, 2021 [cited 2021 Aug 4]. https://www.dshs.texas.gov/IDCU/disease/chagas/Chagas-Disease-Data.aspx

3. Edwards MS, Rench MA, Todd CW, Czaicki N, Steurer FJ, Bern C, et al. Perinatal screening for Chagas disease in southern Texas. J Pediatric Infect Dis Soc. 2015;4:67–70. PubMed https://doi.org/10.1093/jpids/pit056

4. Stimpert KK, Montgomery SP. Physician awareness of Chagas disease, USA. Emerg Infect Dis. 2010;16:871–2. PubMed https://doi.org/10.3201/eid1605.091440

5. Verani JR, Montgomery SP, Schulkin J, Anderson B, Jones JL. Survey of obstetrician-gynecologists in the United States about Chagas disease. Am J Trop Med Hyg. 2010;83:891–5. PubMed https://doi.org/10.4269/ajtmh.2010.09-0543

6. Whitman JD, Bulman CA, Gunderson EL, Irish AM, Townsend RL, Stramer SL, et al. Chagas disease serological test performance in U.S. blood donor specimens. J Clin Microbiol. 2019;57:e01217-19. PubMed https://doi.org/10.1128/JCM.01217-19

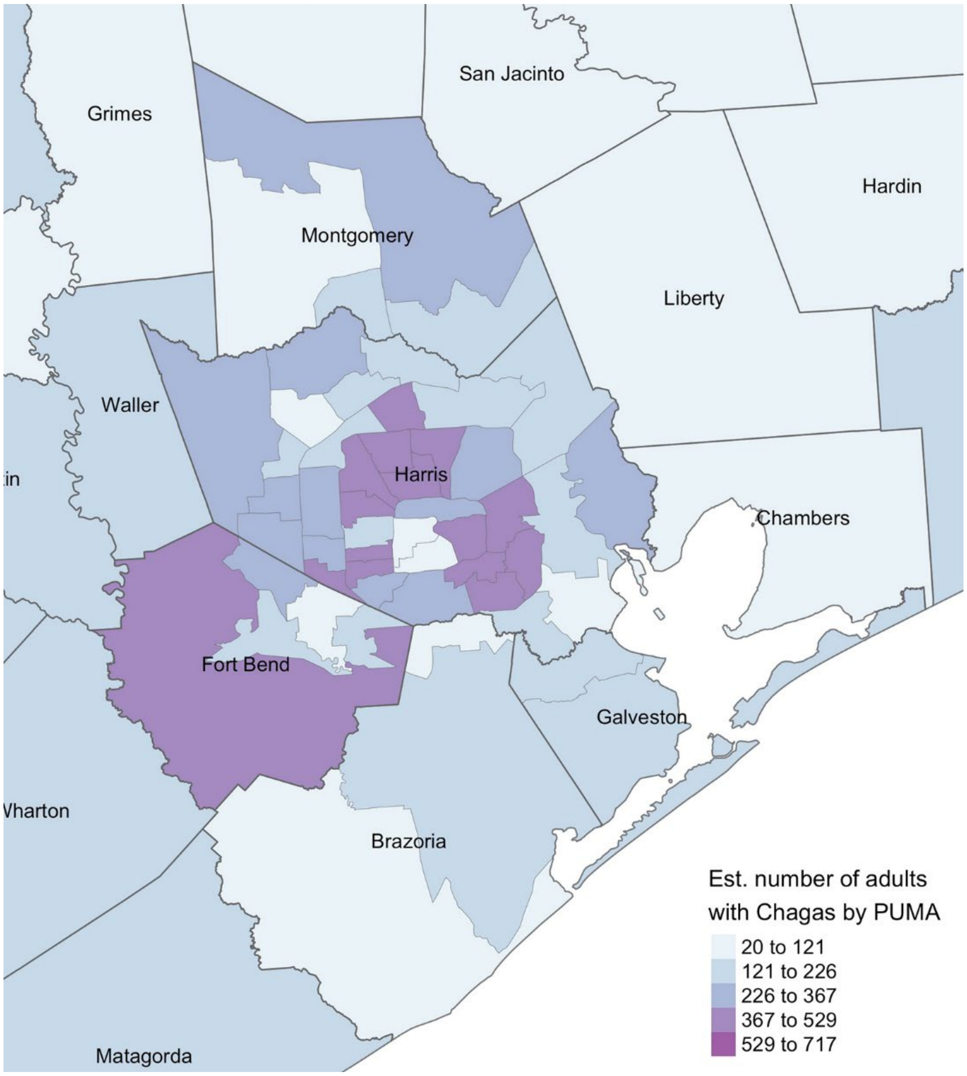

Figure. Map of the metropolitan Houston, Texas, area, showing estimated numbers of adults with Chagas disease. PUMA, Public Use Micro-Area.
