# Supplementary material for: Updated Estimates and Mapping for Prevalence of Chagas Disease among Adults, United States
Source: Emerg Infect Dis. 2022 Jul;28(7):1313–20. doi: 10.3201/eid2807.212221 (PMC9239882; doi:10.3201/eid2807.212221)
Supplement: Appendix 3 — Additional information about Chagas disease in Southern California. [file 21-2221-Techapp-s3.pdf]

Article DOI: https://doi.org/10.3201/eid2807.212221

Updated Estimates and Prevalence of Chagas Disease among Adults, United States

Appendix 3

Southern California

Chagas disease is reportable in Los Angeles County, California (http://publichealth.lacounty.gov/acd/procs/b73/B73Index.htm). We estimate that nearly 45,000 infected persons live in the Los Angeles metro area, the highest burden of any metropolitan area in the United States. This burden includes an estimated 9,400 cardiomyopathy patients and 266 yearly births to infected women. An impressive body of work from the Olive View UCLA Chagas disease group confirms this high disease burden, with substantial numbers of Chagas cardiomyopathy cases diagnosed (*1–4*).

However, contiguous areas stretching south and east show a sprawling pattern of risk, including extensive rural areas in Riverside and Imperial counties (Figure). In rural areas, many of the infected are likely to be agricultural workers, including migrant workers. Although the ACS does not exclude migrant workers, they may be underrepresented in our estimates due to lack of a stable address and potential fears related to participation in a federal survey. These populations will present different challenges to effective screening and experience more marked barriers to healthcare access. Like Texas, Southern California also represents areas of human interaction with *T. cruzi* infected triatomine vectors (*5*). While documented autochthonous cases are rare (*6*,*7*), the extent of inhabited rural areas, such as the eastern desert regions, increases the risk for exposure to infected vectors (*5*,*8*).

The diversity of areas with high Chagas disease prevalence in Southern California also encompasses heterogeneous healthcare systems providing different levels of care. Access to public healthcare systems poses geographic challenges considering the different sizes of catchment areas organized at the county level (e.g., Los Angeles versus Riverside county). A

diagnosis of Chagas disease initiates a significant clinical workup and potentially lifelong management plan, necessitating coordination of clinical and public health approaches.

References

1. Meymandi SK, Forsyth CJ, Soverow J, Hernandez S, Sanchez D, Montgomery SP, et al. Prevalence of Chagas disease in the Latin American-born population of Los Angeles. Clin Infect Dis. 2017;64:1182–8. PubMed https://doi.org/10.1093/cid/cix064

2. Meymandi SK, Hernandez S, Forsyth CJ. A community-based screening program for Chagas disease in the USA. Trends Parasitol. 2017;33:828–31. PubMed https://doi.org/10.1016/j.pt.2017.07.003

3. Traina MI, Hernandez S, Sanchez DR, Dufani J, Salih M, Abuhamidah AM, et al. Prevalence of Chagas disease in a U.S. population of Latin American immigrants with conduction abnormalities on electrocardiogram. PLoS Negl Trop Dis. 2017;11:e0005244. PubMed https://doi.org/10.1371/journal.pntd.0005244

4. Traina MI, Sanchez DR, Hernandez S, Bradfield JS, Labedi MR, Ngab TA, et al. Prevalence and Impact of Chagas disease among Latin American immigrants with nonischemic cardiomyopathy in Los Angeles, California. Circ Heart Fail. 2015;8:938–43. PubMed https://doi.org/10.1161/CIRCHEARTFAILURE.115.002229

5. Hwang WS, Zhang G, Maslov D, Weirauch C. Infection rates of *Triatoma protracta* (Uhler) with *Trypanosoma cruzi* in Southern California and molecular identification of trypanosomes. Am J Trop Med Hyg. 2010;83:1020–2. PubMed https://doi.org/10.4269/ajtmh.2010.10-0167

6. Hernandez S, Flores CA, Viana GM, Sanchez DR, Traina MI, Meymandi SK. Autochthonous transmission of *Trypanosoma Cruzi* in southern California. Open Forum Infect Dis. 2016;3:ofw227. PubMed https://doi.org/10.1093/ofid/ofw227

7. Navin TR, Roberto RR, Juranek DD, Limpakarnjanarat K, Mortenson EW, Clover JR, et al. Human and sylvatic *Trypanosoma cruzi* infection in California. Am J Public Health. 1985;75:366–9. PubMed https://doi.org/10.2105/AJPH.75.4.366

8. Shender LA, Lewis MD, Rejmanek D, Mazet JA. Molecular diversity of *Trypanosoma cruzi* detected in the vector *Triatoma protracta* from California, USA. PLoS Negl Trop Dis. 2016;10:e0004291. PubMed https://doi.org/10.1371/journal.pntd.0004291

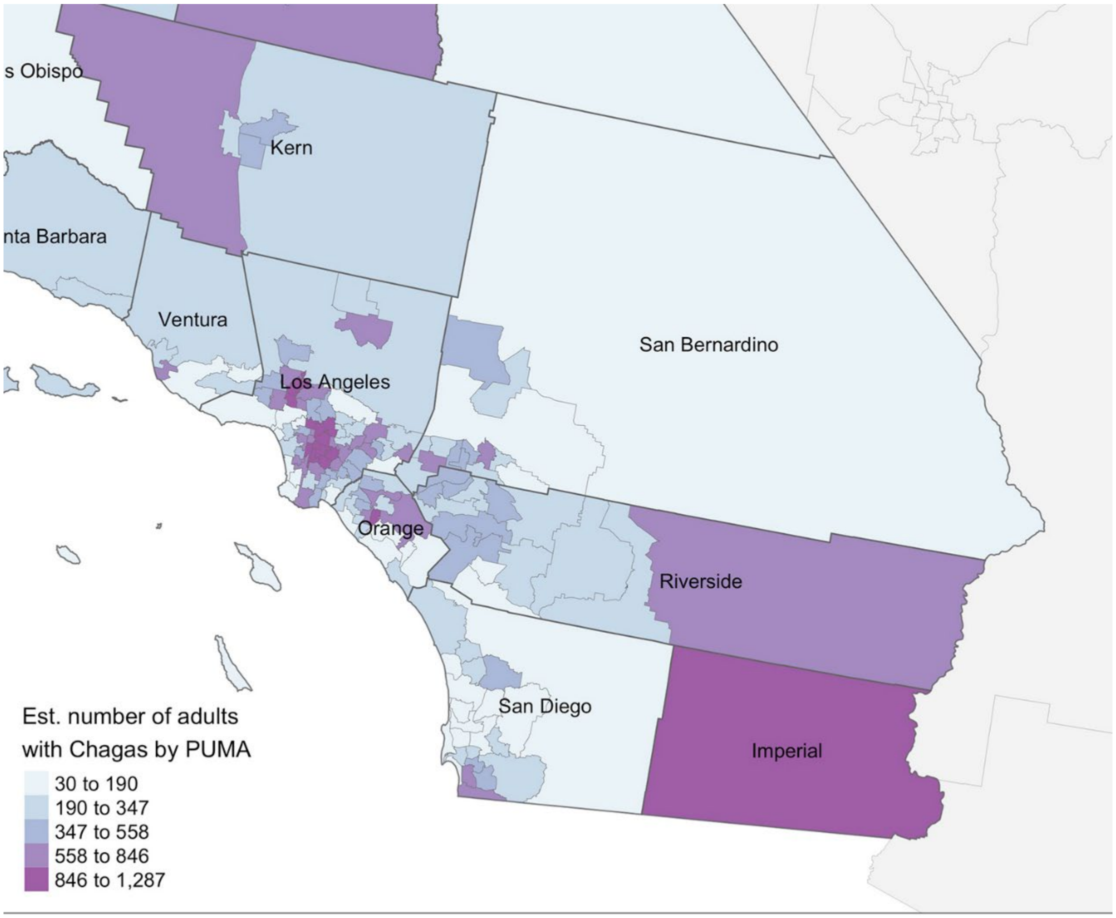

Figure. Map of the Southern California area, showing estimated numbers of adults with Chagas disease. PUMA, Public Use Micro-Area.
