# Supplementary material for: Updated Estimates and Mapping for Prevalence of Chagas Disease among Adults, United States
Source: Emerg Infect Dis. 2022 Jul;28(7):1313–20. doi: 10.3201/eid2807.212221 (PMC9239882; doi:10.3201/eid2807.212221)
Supplement: Appendix 4 — Additional information about Chagas disease in the Washington, DC, metropolitan area. [file 21-2221-Techapp-s4.pdf]

Article DOI: https://doi.org/10.3201/eid2807.212221

Updated Estimates and Prevalence of Chagas Disease among Adults, United States

Appendix 4

Chagas Disease in Washington, DC, Metropolitan Area

We estimate that nearly 18,000 *T. cruzi*–infected persons live in the DC metro area and that 3,400 have Chagas cardiomyopathy. The Latin American immigrant community at risk for Chagas disease resides in a patchwork distribution across the 3 jurisdictions surrounding and within the nation's capital. This immigrant community likely has the highest per-capita prevalence of Chagas disease in the country because of its unusual demographics, with many immigrants from El Salvador and a large high-risk immigrant community from Bolivia in northern Virginia (*1*). The most affected Public Use Micro-Area (PUMA) in the country is found in Fairfax County, with >2,000 estimated infections, most of Bolivian origin, in a total population of 150,000 (Figure). In 8 northern Virginia PUMAs, the prevalence of Chagas disease among Latin American immigrants is >5%, reaching 8.7% in the most affected PUMA.

The most important complicating factor in implementing large-scale screening for Chagas disease in the DC metropolitan area is the lack of easily accessible and affordable healthcare for the at-risk community (*2*,*3*). This is further complicated by the fact that residents with a state-based healthcare plan for the indigent often cross jurisdictional lines for emergency services, and are then unable to receive follow-up at that facility once discharged. County-specific programs designed for those who are not able to purchase insurance further complicate coordination of care. Although a robust federally qualified healthcare network is available in the area, lack of specialty care is particularly important for persons with cardiac disease, and these clinics do not have sufficient resources to afford costly serologic testing and cardiac evaluations (*2*).

Because of high levels of awareness of Chagas disease (*4*), Bolivians participate in screening events and seek out testing far more frequently than other at-risk populations. Awareness is much lower among persons from other countries (*2*,*5*,*6*). Given the costs associated with testing and worry about the potential costs of ongoing care, these persons are frequently unwilling to engage in testing even if they are aware of family members with Chagas disease. The data in these maps illustrate areas where screening will most easily identify *T. cruzi*–infected persons and where educational programming designed for immigrants from the specific countries of origin should be deployed. Screening in both prenatal and cardiac care settings should be accorded high priority given the high risk for disease in this area (*7*). The geographic concentration of those at highest risk can aid these efforts.

References

1. Castro-Sesquen YE, Saldana A, Patino Nava D, Bayangos T, Paulette Evans D, DeToy K, et al. Use of a latent class analysis in the diagnosis of chronic Chagas disease in the Washington metropolitan area. Clin Infect Dis. 2021;72:e303–10. PubMed

2. Forsyth C, Meymandi S, Moss I, Cone J, Cohen R, Batista C. Proposed multidimensional framework for understanding Chagas disease healthcare barriers in the United States. PLoS Negl Trop Dis. 2019;13:e0007447. PubMed https://doi.org/10.1371/journal.pntd.0007447

3. Manne-Goehler J, Reich MR, Wirtz VJ. Access to care for Chagas disease in the United States: a health systems analysis. Am J Trop Med Hyg. 2015;93:108–13. PubMed https://doi.org/10.4269/ajtmh.14-0826

4. Romay-Barja M, Iglesias-Rus L, Boquete T, Benito A, Blasco-Hernández T. Key Chagas disease missing knowledge among at-risk population in Spain affecting diagnosis and treatment. Infect Dis Poverty. 2021;10:55. PubMed https://doi.org/10.1186/s40249-021-00841-4

5. Minneman RM, Hennink MM, Nicholls A, Salek SS, Palomeque FS, Khawja A, et al. Barriers to testing and treatment for Chagas disease among Latino immigrants in Georgia. J Parasitol Res. 2012;2012:295034. PubMed https://doi.org/10.1155/2012/295034

6. Sanchez DR, Traina MI, Hernandez S, Smer AM, Khamag H, Meymandi SK. Chagas disease awareness among Latin American immigrants living in Los Angeles, California. Am J Trop Med Hyg. 2014;91:915–9. PubMed https://doi.org/10.4269/ajtmh.14-0305

7. Forsyth CJ, Manne-Goehler J, Bern C, Whitman J, Hochberg NS, Edwards M, et al. Recommendations for screening and diagnosis of Chagas disease in the United States. J Infect Dis. 2021;225:1601–10. https://doi.org/10.1093/infdis/jiab513

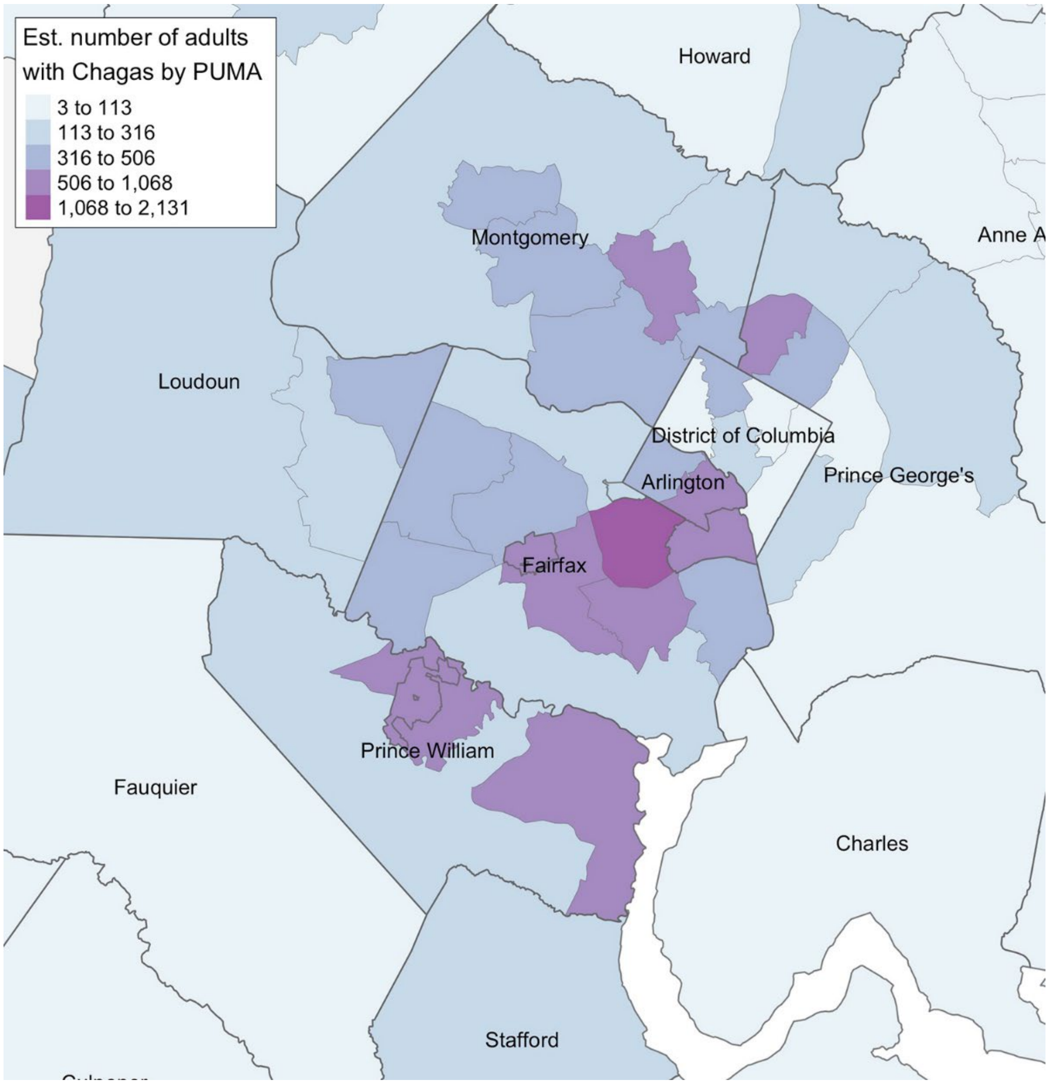

Figure. Map of the metropolitan Washington, DC, area, showing estimated numbers of adults with Chagas disease. PUMA, Public Use Micro-Area.
